# Anti-HPV16 oncoproteins siRNA therapy for cervical cancer using a novel transdermal peptide PKU12

**DOI:** 10.3389/fonc.2023.1175958

**Published:** 2023-06-07

**Authors:** Yan Deng, Yi Song, Quan Du, Chi Chiu Wang, Hu Li, Yi Sui, Yuying Zhang, Tao Tang

**Affiliations:** ^1^ Department of Obstetrics & Gynaecology, Faculty of Medicine, The Chinese University of Hong Kong, Hong Kong, Hong Kong SAR, China; ^2^ The State Key Laboratory of Natural and Biomimetic Drugs, School of Pharmaceutical Sciences, Peking University, Beijing, China; ^3^ Li Ka Shing Institute of Health Science, and School of Biomedical Sciences, The Chinese University of Hong Kong, Hong Kong, Hong Kong SAR, China; ^4^ Department of Gynecology, Pan Yu Central Hospital, Guangzhou, China; ^5^ Cancer Institute, Panyu Central Hospital, Guangzhou, China; ^6^ Department of Nutrition, The First Affiliated Hospital of Sun Yat-sen University, Guangzhou, China; ^7^ Department of Obstetrics and Gynaecology, Shenzhen Longhua Maternity and Child Healthcare Hospital, Shenzhen, China

**Keywords:** transdermal peptide, HPV, siRNA, cervical cancer, RNA-sequencing, signaling pathway

## Abstract

In this study, an innovative transdermal peptide, #PKU12, was developed based on transdermal peptide TD-1, and the anti-tumor effect of PKU12-based siRNA against HPV was investigated *in vivo*. Furthermore, transcriptome differences between PKU12 + siRNA treatment and control groups were compared to assess treatment effects. The top five upregulated and downregulated genes identified by RNA sequencing were further subjected to survival analysis. The present study, for the first time, showed that this novel peptide could enhance the transdermal delivery of the siRNA targeting HPV16 L1, E6, and E7. PKU12-based siRNA delivery significantly repressed the mRNA expression levels of HPV16 L1, E6, and E7 in the SiHa xenograft tumors and attenuated tumor growth as well. The RNA-sequencing results showed that a total of 586 DEGs were detected in the PKU12 + siRNA-treated tumor tissues compared to the control tumor tissues. The GSEA analysis revealed that DEGs were inversely associated with the HIF-1 signaling pathway, the TNF signaling pathway, the AGE-RAGE signaling pathway, the NF-kappa B signaling pathway, ferroptosis, the IL-17 signaling pathway, ovarian steroidogenesis, and rheumatoid arthritis. Further functional enrichment analysis revealed that DEGs were significantly enriched in several key pathways, including cytokine–cytokine receptor interaction, the TNF signaling pathway, and the IL-17 signaling pathway. High expression of MYH1, MYH4, FGG, DEPP1, and ZBTB16 was associated with shorter overall survival of patients with cervical cancer; high expression of SULT1E1, RAB3C, CXCR3, and PROX2 was associated with longer overall survival of patients with cervical cancer. In conclusion, the transdermal peptide PKU12 is potentially a good candidate for a siRNA delivery vehicle for the treatment of cervical cancer.

## Introduction

Cervical cancer is one of the most common malignant tumors that endanger women's health. According to WHO reports, there are about 604,000 new cervical cancer cases in the world every year, mainly in developing countries. Among them, China accounts for approximately 18% of this burden (1, 2). At present, the treatment of cervical cancer is mostly based on surgery and radiation therapy, combined with chemotherapy, which will inevitably bring pain to patients (3). These treatments may cause irreparable harm. Thus, it is an important and urgent task to find new technologies and methods to prevent and treat cervical cancer.

With the rapid development of molecular biology, the emergence of RNA interference technology has opened a new way for the prevention and treatment of various diseases, such as virus-induced diseases and tumors. RNA interference refers to some small double-stranded RNA (dsRNA) that can efficiently and specifically block the expression of specific genes, promote mRNA degradation, and induce cells to show the phenotype of specific gene deletion. RNAi is revolutionizing the field of functional genomics and will revolutionize the pace of research in this field because it can be used as a simple and effective genetic tool to replace gene knockouts (4). SiRNAs have been extensively used to silence cancer-related gene targets. From a clinical perspective, the siRNA approach may represent a more suitable and safe strategy for the management of cervical cancer when compared to commonly used radiation or chemo-radiation therapy (5).

Human papillomavirus (HPV) is a group of viruses that includes more than 100 types. Approximately 99% of cervical carcinoma patients are diagnosed with HPV infection (6). Among them, HPV16 and HPV18 are the most important types, and they cause approximately 70% of cervical carcinoma cases (7). The genomes of all HPVs contain eight ORFs, and the ORF can be divided into three functional parts: the early region, which encodes proteins (E1–E7); the late region, which encodes structural proteins (L1–L2); and a largely non-coding part, in which the E6 and E7 proteins target several cell cycle regulators, primarily p53 and pRB, respectively (8). L1 is the major structural protein required for virus assembly (9). Since cervical cancer is mainly caused by HPV, HPV is the most important etiological agent. Importantly, the HPV oncogene is exogenous. Therefore, siRNA treatment targeting HPV L1, E6, and E7 would not affect the host genes. On the other hand, for Stage 0 and Stage I cervical cancer, abnormal cells or a very small amount of cancer cells are found only in the innermost tissue of the cervix, and local treatment may be enough and appropriate for the patients. In terms of indications, silencing E6 and E7 is mainly for the treatment of cervical cancer, while silencing L1 is for the treatment of cervical inflammation.

Topical use of therapeutic siRNA has been increasingly studied due to the importance of treating skin diseases, topical application to treat tumors, and improving skin properties. However, application of naked siRNA does not exert strong therapeutic effects due to its low delivery efficiency to target tissues and cells and various barriers. Traditionally, siRNA delivery is assisted by liposomes. However, the lipophilic analogs are sometimes toxic to cells (10, 11). In recent years, biologically inspired peptides, discovered using the phage display technique, have been used to facilitate the delivery of macromolecular drugs into the skin (12). These short peptides make it possible to deliver siRNAs *via* the skin or mucosa to cells (13, 14). Transdermal Peptide TD-1 is the short synthetic peptide ACSSSPSKHCG, identified by *in vivo* phage display, which facilitates efficient transdermal protein drug delivery through intact skin. Co-administration of TD-1 and insulin to the abdominal skin of diabetic rats resulted in elevated systemic levels of insulin and suppressed serum glucose levels for at least 11 h. In another study, TD-1 delivery of anti-GAPDH siRNA reduced the level of GAPDH in the back and footpad skin of rats (12, 15).

In this study, we developed an innovative transdermal peptide called #PKU12, which is a single amino acid variant of TD-1. We used a nude mouse xenograft model to investigate the anti-tumor effect of PKU12-based siRNA against HPV-delivered cervical cancer. Transcriptome changes occur during the development of cancer, and a specific treatment can significantly alter the transcriptome of cancer cells, which serves as an indicator for evaluating cancer treatment. Additionally, we observed transcriptome differences between the treatment and control groups to assess treatment effects.

## Materials and methods

### Ethics statement

SD rats and BALB/c athymic mice were used in the study. All experimental protocols were approved by the Animal Experimentation Ethics Committee of Peking University and the Chinese University of Hong Kong.

### Preparation of PKU12

To efficiently construct a single amino acid variant TD-1 polypeptide expression library, a single amino acid variant cloning vector library was constructed. On this basis, the original sequence of TD-1 reported in the literature was used as a template to construct a TD-1 polypeptide sequence library containing single amino acid variations in the T vector and then introduce the polypeptide library into a phage expression plasmid. By using mouse models, systematic screening (blue-white screening) was conducted to obtain a phage library to identify phages with higher transdermal effects. The details were as follows: 100 μl of bacteriophage in PBS with 15% glycerol was topically applied over a 3 cm^2^ area on the abdominal skin of the animals. After 1–2 h, 5–10 μl of blood was taken from the tail vein, immediately mixed with 200 μl of ER2738-competent cells that had been grown for 4 h in LB medium at 37 °C, 120 rpm, and incubated at 37 °C, 120 rpm for half an hour. The screening plate is preheated half an hour in advance and then smears a total of 67 μl of solution/plate according to the ratio of Xgal:IPtg:TET = 40:7:20 μl. When the surface of the culture plate is dry, the bacterial solution obtained before is plated on the screening culture plate for blue-white screening and placed at 37 °C overnight. For blue-white screening, the phage expression plasmid contains a segment of the lacZ α gene, which will be taken up by ER2738-competent cells. LacZ α gene in ER2738-competent cells encodes β-galactosidase. β-galactosidase can hydrolyze X-gal, resulting in 5-bromo-4-chloro-indoxyl, which dimerizes to form a blue pigment. Scraping to obtain all monoclonal plaques (blue pigment) to form a subclonal library, that is, to complete a round of screening. Using the obtained subcloned library, repeat the above steps and continue to complete the second and third rounds of screening. Extract the DNA of the positive clone obtained by the third round and do the bacterial liquid PCR and sequencing verification.

Blue-white screening (the same mouse models as above) was also employed to compare the transdermal activities of TD1, and the selected sequence was named PKU12. In the screening, TD-1 was used as the positive control; blank (no phage was added during incubation) and GLP-1 (which has no transdermal activity) were used as the negative control.

### SiRNAs

The 3′-labeled Cy5 siRNA, 5′ cholesterol human HPV16 L1, E6 and E7 siRNA, and nonsense siRNA were purchased from RioboBio (Guangzhou, China). The sequences of the siRNAs are as follows:

L1 5’- CCCAGUAUCUAAGGUUGUAdTdT-3’ (sense)
5’- UACAACCUUAGAUACUGGGdTdT-3’ (antisense)
E6 5’- GUACUGCAAGCAACAGUUAdTdT-3’ (sense)
5’-UAACUGUUGCUUGCAGUACdTdT-3’ (antisense)
E7 5’-GAUUUGCAACCAGAGACAAdTdT-3’ (sense)
5’-UUGUCUCUGGUUGCAAAUCdTdT-3’ (antisense)
Nonsense 5’ (universal NC siRNA sequence, siN0000001-1-5, siR NC #1, RioboBio)

SiRNA and PKU12 were mixed and dissolved in RNase free water in the mole ratio of 100:1 to 3,000:1.

### Peptide and siRNA penetration in skin

The hair of an adult SD rat in a 2 cm × 2 cm area on the abdomen was carefully trimmed with scissors. Chemically synthesized PKU12 was mixed with Cy5-labeled siRNA (100 ng siRNA and 1 mg PKU12 in 0.1 g cosmetic cream). The cosmetic cream is composed of several key ingredients, including water, glycerin, glyceryl stearate, mineral oil, and dimethicone. Glycerin works to moisturize and retain water, while glyceryl stearate functions as an emulsifier. Mineral oil is beneficial in locking moisture into the skin, and dimethicone serves as a gentle yet potent moisturizing ingredient. It was then topically applied to the trimmed abdominal skin of the rats and wrapped with a plastic wrap. An hour later, the mice were sacrificed, and the skin was isolated and washed with PBS. The samples were placed in 4% paraformaldehyde overnight, frozen in OCT compound, and sectioned at a thickness of 15 μm. The slides were washed for 5 min prior to staining with 5 μg/ml Hoechst 33342 (Invitrogen) for 5 min. The slides were washed again for 5 min and then allowed to dry completely at room temperature in the dark. All samples were imaged with a confocal microscope (Leica TCS SP8 WLL Inverted Confocal Microscope).

### Cell culture

The cervical cancer cell line SiHa (American Type Cell Culture Collection, VA, USA) was cultured in Dulbecco’s modified Eagle’s medium (DMEM, Hyclone, GE Health Care, USA) supplemented with 10% fetal bovine serum. The cells were incubated in a humidified atmosphere with 5% CO_2_ at 37 °C.

### Test of therapeutic effect of HPV16 siRNAs delivered by PKU12 in cervical tumor xenograft mice model

Eighteen female BALB/c athymic mice will be anesthetized with ketamine (75 mg/kg) or xylazine (10 mg/kg), and then 1 × 10^7^ cervical cancer cells (SiHa) will be injected into the back flank of the mice by subcutaneous injection using a microsyringe fitted with a 30G needle. Fourteen days after injection, the mice were assigned to three groups: 1) PKU12 and HPV16L1, E6, and E7 siRNA formula; 2) PKU12 and nonsense siRNA as a control; and 3) HPV16L1, E6, and E7 siRNA formula alone, n = 6 for each group. The siRNA and transdermal peptides were stored at −20 °C in the form of lyophilized powder. When using 10 μg siRNA and 1 mg PKU12 per animal, the solution required for smearing is 20 μl, that is 10 μg siRNA/mg PKU12 in 20 μl of RNase free water. The prepared solution is stored at 4 °C and used within 48 h. The PKU12 and siRNA formula solution was aspirated with a pipette tip and applied to the tumor site once a day for 10 days. The tumor volume and body weight of mice were measured every three days. Then the mice were terminated, and the weight of the cervical tumor xenograft was measured. RNA was extracted for qRT-PCR/RNA-seq. Four samples from the control group and four samples from the PKU12, HPV16L1, E6, and E7 siRNA formula groups were randomly selected for RNA-Seq, and the samples were sequenced individually.

### Quantitative real-time PCR

Total RNA was extracted by homogenization in Trizol reagent (Invitrogen, Cat. No. 15596-018), followed by chloroform extraction and isopropanol precipitation. Approximately 1,000 ng of total RNA were reverse-transcribed to cDNA using the commercial protocol (PrimeScript™ RT Master Mix, TAKARA). qRT-PCR was performed using SYBR® Premix Ex Taq ™ (TAKARA) and an ABI Prism 7900HT Sequence Detection System (Applied Biosystems). GAPDH was used as an internal control, and the relative expression of target RNA was determined by 2^−ΔΔCt^ method. The primer sequences for the qRT-PCR analysis are shown in **Table 1**.

**Table 1:** Primer sequences for qRT-PCR analysis.

Gene	Forward (5’-3’)		Reverse (5’-3’)
HPV16 L1	CCCAAACGCACCGAATAGTTAC		ATTCCAATTCCCCTGCAAACT
HPV16 E6	CACAGGAGCGACCCAGAAAG		GCATAAATCCCGAAAAGCAA
HPV16 E7	ATGCATGGAGATACACCTAC		TTATGGTTTCTGAGAACAGA
GAPDH	AGGGCTGCTTTTAACTCTGGT		CCCCACTTGATTTTGGAGGGA

### RNA-sequencing analysis

Total RNA was extracted using Trizol reagent (Invitrogen, Cat. No. 15596-018) according to the instructions. The concentration of RNA was measured using a NanoDrop 2000 spectrophotometer (Thermo Fisher, USA), and the integrity of RNA was determined using an Agilent 5400 Bioanalyzer (Agilent Technologies, USA). Samples with a RIN value greater than 4 were used for subsequent library preparation and sequencing. The mRNA libraries were prepared according to the instructions provided in the NEBNext Ultra RNA Library Prep Kit for Illumina (NEB, Cat. No. E7530L) kit. Poly(A)-tailed mRNA was enriched using oligo(dT) magnetic beads, and the mRNA was fragmented using divalent cations in NEB Fragmentation Buffer. Random primers were used to synthesize the first-strand cDNA using the fragmented mRNA as a template in the M-MuLV reverse transcriptase system. The RNA strand was then degraded using RNaseH, and the second strand of cDNA was synthesized using DNA polymerase I and dNTPs as raw materials. The purified double-stranded cDNA was end-repaired, adenylated, and ligated with sequencing adapters. cDNA fragments of approximately 200 bp were selected using AMPure XP beads (Beckman Coulter, Cat. No. A63881), PCR amplified, and purified again using AMPure XP beads to obtain the final library. After library construction, the library was initially quantified using a Qubit 2.0 fluorometer (Thermo Fisher, USA) and diluted to 1.5 ng/μl. The insert size of the library was determined using an Agilent 2100 Bioanalyzer (Agilent Technologies, USA), and qRT-PCR was used to accurately quantify the effective concentration of the library (library effective concentration >2 nM) to ensure library quality. After quality inspection, the library was sequenced using the Illumina NovaSeq 6000 sequencer (Illumina, USA) with the PE150 strategy. After sequencing, Cutadapt v1.18 software was used to remove adapter sequences and filter out low-quality reads. The filtered reads were aligned to the reference sequence using STAR v2.7.1a software. Quantitative analysis was performed using featureCounts v2.0.1 software, and gene expression quantification was measured in FPKM. Differential expression analysis was performed using edgeR v3.28.1, with a screening threshold set at p-value <0.05 and |log2(FoldChange)| >1 (a specific screening threshold can be found in the report), with genes having log2(FoldChange) >1 considered upregulated genes and those with log2(FoldChange) <1 considered downregulated genes. The heatmap clustering of differentially expressed genes was generated using the R (R.3.6.0) package. GO enrichment analysis of differentially expressed genes was performed using topGO v2.38.1, and Fisher’s exact test was used as the statistical test. KEGG pathway enrichment analysis of differentially expressed genes was performed using clusterProfiler v3.14.3, and the hypergeometric test was used as the statistical test. GSEA analysis of the GO and KEGG pathway datasets was performed separately using clusterProfiler software.

### Kaplan–Meier survival curve analysis

The Kaplan–Meier plotter (http://kmplot.com/analysis/) was used to analyze the relationship between gene expression and survival rates in cervical cancers based on hazard ratios (HR) and log-rank P-values. The Kaplan–Meier curves were plotted using 304 patients with cervical cancer, and the database was acquired from acquired from The Cancer Genome Atlas (TCGA) repository (16).

### Statistical analysis

All data analysis was performed using GraphPad Prism Version 6.0 (GraphPad Software, La Jolla, USA). Data are presented as the mean ± standard deviation. For tumor tissue gene expression and tumor weight, statistical comparisons between groups were made by one-way ANOVA followed by Bonferroni’s multiple comparison. For tumor growth and body weight, statistical comparisons between groups were made by two-way ANOVA followed by Bonferroni’s multiple comparison. *P* <0.05 was considered to be statistically significant.

## Results

### Generation of transdermal peptide PKU12

**Figure 1:**
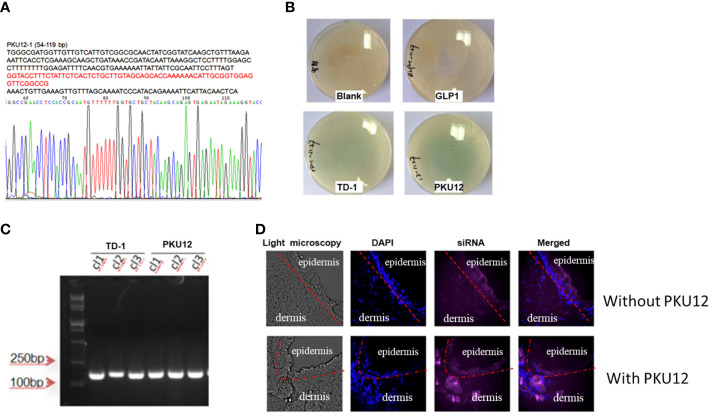
Generation of PKU12 peptide and validation of its ability to enhance dermal penetration. **(A)** Sequences of PKU12. **(B)** Blue-white screening to determine the transdermal ability of PKU12. **(C)** PCR analysis of the colonies. **(D)** Fluorescent staining of the transdermal-delivered siRNA.

After three rounds of blue-white screening, we extracted the DNA of the positive clones and performed the sequencing, getting the peptide sequence ACSSTKKHCG (PKU12) (**Figure 1A**). To verify the transdermal effect of PKU12, we conducted another blue-white screening. On culture plates, plaques are shown in blue. More blue plaques indicate better transdermal ability. The PKU12 plate showed more blue plaques than the TD-1 plate, indicating better transdermal ability than TD-1. On the blank and GLP plates, there was no plaque (**Figure 1B**). The colonies were further validated by PCR (**Figure 1C**). Furthermore, we investigated the ability of PKU12 to enhance the dermal penetration of Cy5-labeled siRNA. The blue signal represents the nuclear, and the purple signal indicates the siRNA. The epidermis is the outermost of the three layers that make up the skin, and the inner layers are the dermis. PKU12-assisted siRNA across the epidermis. Fluorescently tagged siRNA was distributed throughout the epidermis and dermis, and the siRNA exhibited cytoplasmic perinuclear localization, suggesting cellular uptake. Without PKU12, the siRNA cumulated mainly on the skin surface (**Figure 1D**).

### Anti-tumor effects of PKU12-based siRNA delivery in SiHa cell xenograft

**Figure 2:**
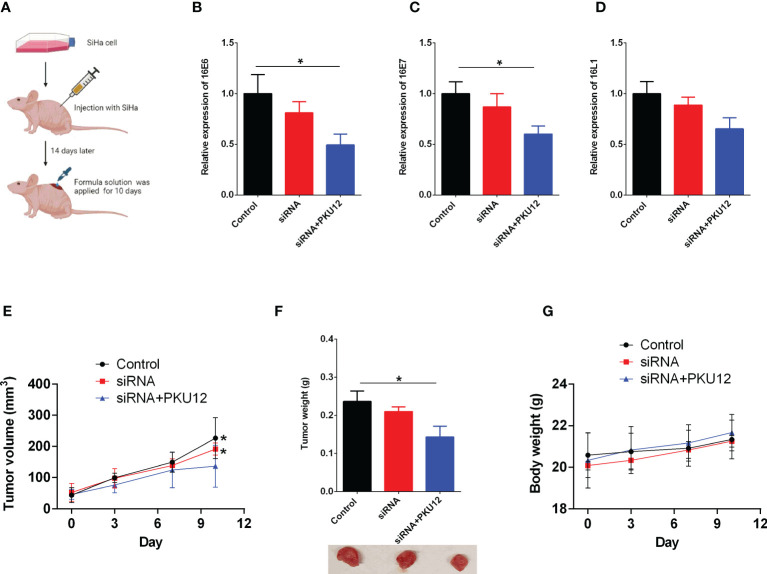
The *in vivo* anti-tumor effects of PKU12-based siRNA delivery in SiHa cells. **(A)** Schematic drawing of the *in vivo* study. **(B–D)** The effects of siRNA delivery and PKU12-based siRNA delivery on the **(B)** 16E6 expression, **(C)** 16E7 expression and **(D)** 16L1 expression in tumor tissues were determined by qRT-PCR. **(E, F)** The effects of siRNA delivery and PKU12-based siRNA delivery on the tumor volume and tumor weight were evaluated. **(G)** The effects of siRNA delivery and PKU12-based siRNA delivery on the body weight were evaluated. N = 6. For tumor tissure gene expression and tumor weight, statistical comparisons between groups were made by one-way ANOVA followd by Bonferroni’s multiple comparison. For tumor growh and body weight, statistical comparisons between groups were made by two-way ANOVA followd by Bonferroni’s multiple comparison. The significant difference between different treatment groups is indicated as *P < 0.05 and **P < 0.01 when compared with PKU12+siRNA group.

The expression levels of HPV16 E6, E7, and L1 in tumor tissues were measured. PKU12 and siRNA treatment showed a significant suppressive effect on the expression levels of E6, E7, and L1. The tumor weight in three groups was measured after being sacrificed. PKU12 and siRNA treatments showed an inhibitory effect on tumor growth. SiRNA alone, however, had no marked effects on E6, E7, or L1 silencing or tumor growth (**Figures 2A–F**). The body weight of mice in three groups was measured at days 0, 3, 7, and 10 (treatment days). There were no significant differences between these groups (**Figure 2G**). The results indicated that PKU12 facilitated siRNAs crossing the dermis layer, playing a role in E6, E7, and L1 silencing, while siRNA alone cannot cross the skin and exert its function. Therefore, PKU12 delivered siRNA against HPV inhibited tumor growth in the nude mouse xenograft model. These treatments have no effect on the body weight of mice.

### Quantification of gene expression

**Figure 3:**
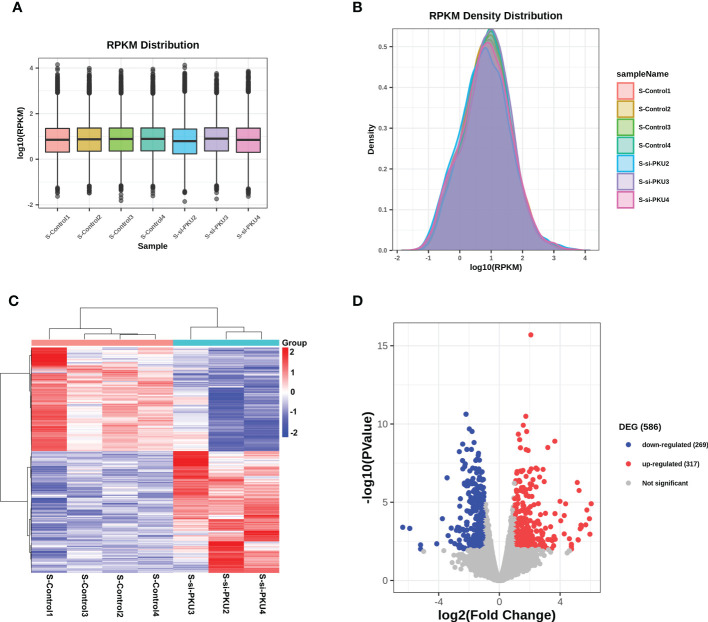
RNA-sequencing analysis of the differentially expressed genes in tumor tissues between control group and PKU12-based siRNA delivery group. **(A)** RPKM distribution of the RNA-seq data. **(B)** RPKM density distribution of the RNA-seq data. **(C)** Heatmap illustrates the differentially expressed genes in the tumor tissues between control group and PKU12-based siRNA delivery group. **(D)** Volcano plot illustrates the differentially expressed genes in the tumor tissues between siRNA delivery group and PKU12-based siRNA delivery group.

RNA-Seq was used to detect the transcriptome profile of the xenografts in both the PKU12 plus siRNA formula group and the control group. The overall gene expression levels of all samples in these two groups were comparable, with no significant differences (**Figures 3A, B**). A hierarchical clustering analysis of differentially expressed genes (DEGs) is shown in **Figure 3C**. The volcano plot results identified 586 DEGs (317 upregulated and 269 downregulated) (**Figure 3D**).

### GSEA analysis

**Figure 4:**
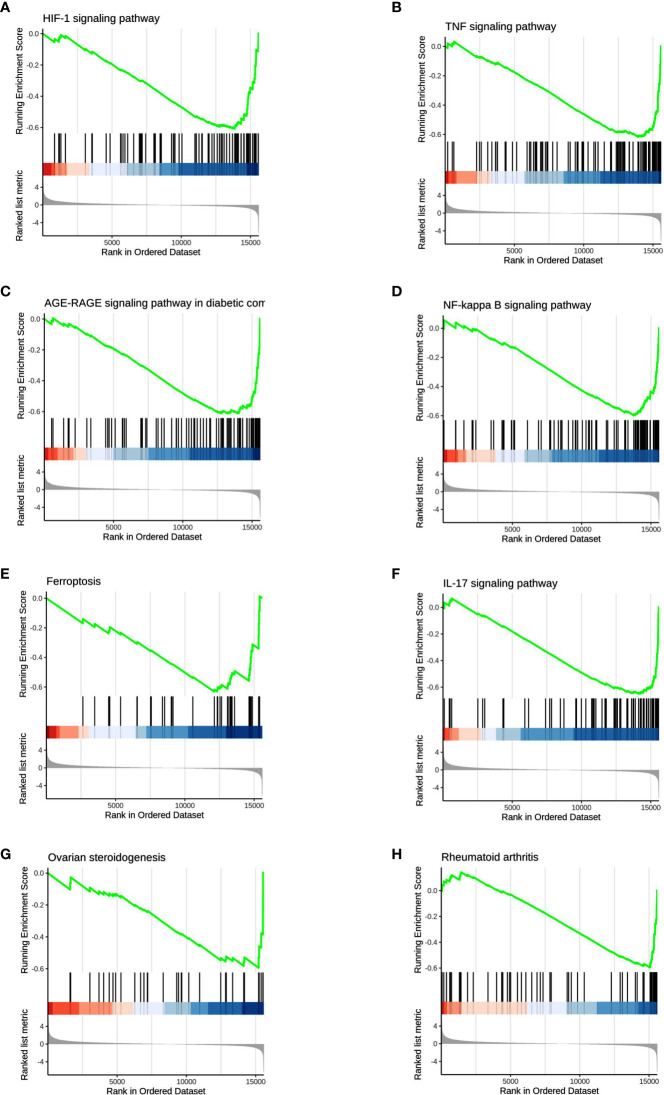
GSEA analysis of the DEGs. The GSEA analysis showed that the DEGs were associated with **(A)** HIF-1 signaling pathway, **(B)** TNF signaling pathway, **(C)** AGE-RAGE signaling pathway, **(D)** NF-kappa B signaling pathway, **(E)** ferroptosis and **(F)** IL-17 signaling pathway, **(G)** ovarian steroidogenesis and **(H)** rheumatoid arthritis.

We used GSEA, a pathway enrichment method, to evaluate RNA-Seq data at the level of gene sets. We found 115 gene sets that were significantly enriched for differentially expressed genes in the PKU12 plus siRNA treatment group compared with the control group (**Supplementary Table S1**). Of these, 112 gene sets were downregulated, and three gene sets were upregulated in the PKU12 plus siRNA treatment group. The top eight enriched gene sets are: HIF-1 signaling pathway, TNF signaling pathway, AGE-RAGE signaling pathway in diabetic complications, NF-kappa B signaling pathway, ferroptosis, IL-17 signaling pathway, ovarian steroidogenesis, and rheumatoid arthritis (**Figure 4**).

### GO and KEGG pathway enrichment analysis

**Figure 5:**
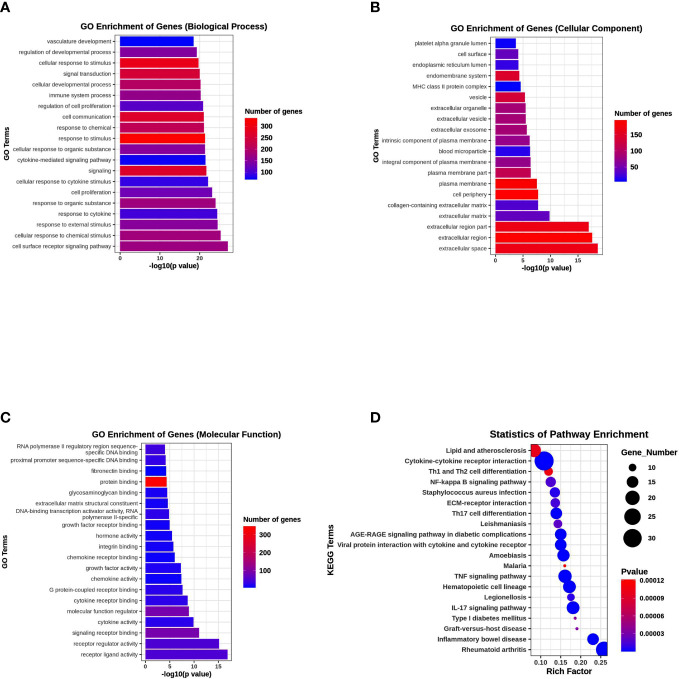
Functional enrichment analysis of DEGs. **(A)** GO enrichment analysis of DEGs in biological process. **(B)** GO enrichment analysis of DEGs in cellular component. **(C)** GO enrichment analysis of DEGs in molecular function. **(D)** KEGG pathway enrichment analysis of DEGs.

To elucidate the possible functional significance of the observed changes in transcripts between the PKU12 plus siRNA formula and control groups, GO and KEGG analysis were performed. The enriched pathways with the most significant top 20 ones with a lower p-value are shown. As shown in **Figures 5A–C**, differentially expressed transcripts had GO annotations to biological processes, cellular components, and molecular functions. Cellular response to stimulus, signal transduction, extracellular region, extracellular region, and protein binding were significantly enriched. For KEGG analysis, cytokine–cytokine receptor interaction, TNF signaling pathway, IL-17 signaling pathway, and rheumatoid arthritis were significantly enriched (**Figure 5D**).

### Prognostic significance of top down/upregulated transcripts expression in cervical cancer

**Figure 6:**
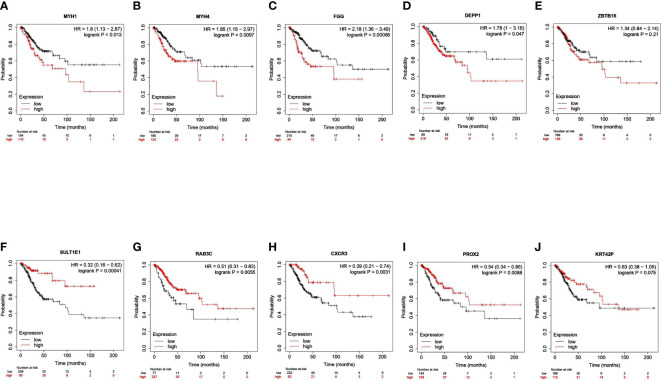
Survival analysis of top 10 DEGs in patients with cervical cancer. The association between overall survival of patients with cervical cancer and the expression of **(A)** MYH1, **(B)** MYH4, **(C)** FGG, **(D)** DEPP1, **(E)** ZBTB16, **(F)** SULTIEI, **(G)** RAB3C, **(H)** CXCR3, **(I)** PROX2 and **(J)** KRRT42P was analyzed using Kaplan-Meier plotter tool.

We identified a total of 586 DEGs, of which MYH1, MYH4, FGG, DEPP1, and ZBTB16 were the top downregulated transcripts. SULTIEI, RAB3C, CXCR3, PROX2, and KRT42P were the top upregulated transcripts (**Table 2**, **Supplementary S1**-**S5**). Next, we analyzed the prognostic value of these expressions in cervical cancer using the Kaplan–Meier plotter database. Based on the hazard ratios (HR) and log-rank P-values, low MYH1, MYH4, FGG, DEPP1, and ZBTB16 expression was associated with a better prognosis. On the other hand, high SULTIEI, RAB3C, CXCR3, PROX2, and KRT42P expression was associated with a better prognosis in cervical cancer (**Figure 6**). This indicated that PKU12-delivering siRNA treatments significantly changed the transcripts of the tumors, and the change was associated with a better prognosis.

**Table 2:** The top down/up regulated transcripts.

	logFC	P value	stat	Gene Ontology	KEGG pathway	Function
MYH1	-6.37135	0.000399	down-regulated	microfilament motor activity; nucleotide binding; cytoskeletal motor activity; actin binding; protein binding	/	Required for proper formation and/or maintenance of myofibers
MYH4	-5.19673	0.009729	down-regulated	microfilament motor activity; nucleotide binding; double-stranded RNA binding; cytoskeletal motor activity	/	Muscle contraction
FGG	-3.00107	0.024318	down-regulated	signaling receptor binding; structural molecule activity; protein binding;	Complement and coagulation cascades (S1)	Guide cell migration during re-epithelialization
DEPP1	-2.944	0.004448	down-regulated	autophagy; regulation of autophagy	/	Regulation of autophagy
ZBTB16	-2.83536	0.000617	down-regulated	RNA polymerase II cis-regulatory region sequence-specific DNA binding; transcription corepressor binding; DNA binding	Transcriptional misregulation in cancer (S2)	Acts as a transcriptional repressor
SULT1E1	4.273896	0.001056	up-regulated	aryl sulfotransferase activity; protein binding; sulfotransferase activity	Steroid hormone biosynthesis (S3)	Play a role in estrogen homeostasis and gut microbiota-host metabolic interaction
RAB3C	4.617507	0.012879	up-regulated	nucleotide binding; GTP binding; protein binding	/	Protein transport
CXCR3	4.743816	0.006685	up-regulated	chemokine receptor activity; protein binding	Chemokine signaling pathway (S4); Cytokine-cytokine receptor interaction (S5)	Promotes cell chemotaxis response; mediates the proliferation, survival and angiogenic activity of human mesangial cells
PROX2	5.20222	0.002615	up-regulated	DNA-binding transcription factor activity, RNA polymerase II-specific; RNA polymerase II cis-regulatory region sequence-specific DNA binding	/	Transcription regulator
KRT42P	5.769005	3.14E-05	up-regulated	/	/	Pseudogene

## Discussion

The present study showed for the first time that a novel peptide (PKU12) could facilitate the transdermal delivery of siRNA targeting HPV16 L1, E6, and E7. Our results showed that PKU12-based siRNA delivery significantly repressed the mRNA expression levels of HPV16 L1, E6, and E7 in the SiHa xenograft tumors, thus attenuating tumor growth. The RNA-sequencing results showed that a total of 586 DEGs were detected in the PKU12 + siRNA-treated tumor tissues as compared to the control tumor tissues. The GSEA analysis revealed that DEGs were inversely associated with the HIF-1 signaling pathway, the TNF signaling pathway, the AGE-RAGE signaling pathway, the NF-kappa B signaling pathway, ferroptosis, the IL-17 signaling pathway, ovarian steroidogenesis, and rheumatoid arthritis. Further functional enrichment analysis revealed that DEGs were significantly enriched in several key pathways, including cytokine–cytokine receptor interaction, the TNF signaling pathway, and the IL-17 signaling pathway. The top five upregulated and downregulated DEGs were further subjected to survival analysis.

Recently, peptide-mediated transdermal delivery of siRNA has emerged as a promising approach to treating various diseases. Tsu et al. developed a skin penetrating and cell-entering (SPACE) peptide and demonstrated that SPACE peptide, when conjugated to cargoes such as small molecules and proteins, was able to facilitate their penetration across the stratum corneum into the epidermis and dermis (14). Uchida et al. demonstrated that Tat and AT1002 peptides could accelerate transdermal siRNA delivery both widely and effectively (13); topical skin application of anti-NF-κB (RelA) siRNA with STR-CH2R4H2C and AT1002 improved atopic dermatitis-like symptoms in model mice (17). Ruan et al. also designed a new and safe fusion peptide carrier, SPACE-EGF, to improve the skin and cell penetration functions of the siRNAs and their targeting ability to B16 cells (18). The cationic cyclopeptide has also been developed to enhance drug delivery. Chang et al. developed a novel peptide (TD-34: ACSSKKSKHCG) based on the sequence of TD-1 and found that TD-34 exhibited enhancement activity on insulin transdermal delivery (19). Further mechanistic studies revealed that TD-34 had the potential to enhance paracellular delivery of insulin across Caco-2 cell monolayers by loosening tight junctions reversibly (20). In addition, Tian et al. also synthesized a novel cyclopeptide (DLCC-2: KWSSKKSKHCG-NH2) and found that DLCC-2 could enhance the transdermal route of DLCC-2 (21). In the present study, we designed a cationic cyclopeptide (PKU12) based on TD-1. Our results showed that PKU12 has better transdermal ability than TD-1. Furthermore, *in vivo* studies revealed that PKU12-based delivery of siRNAs targeting HPV16 L1, E6, and E7 could enhance anti-tumor activity in the mouse xenograft model of SiHa cells.

To further clarify the anti-tumor activities of PKU-based delivery of siRNAs, we performed an RNA-sequence analysis in the tumor tissues, identifying a total of 586 DEGs that were detected in the PKU12 + siRNA-treated tumor tissues as compared to the control tumor tissues. Our GSEA results revealed that the DEGs were inversely correlated with several cancer-related signaling pathways such as HIF-1 signaling, TNF signaling, NF-kappa B signaling, ferroptosis, and IL-17 signaling, which was in agreement with the functional GO and KEGG pathway enrichment analysis. The role of HIF-1 signaling in cervical cancer has been reported in various studies. Lu et al. showed that HIF-1 could facilitate cervical cancer progression in HPV16 transgenic mice (22). Overexpression of HPV16 oncoproteins could enhance HIF-1 alpha protein accumulation and vascular endothelial growth factor expression in human cervical carcinoma cells (23). Further studies also showed that HPV16 E6 contributed to the HIF-1α-induced Warburg effect by attenuating the von Hippel–Lindau tumor suppressor-HIF-1α interaction (24). Our results also demonstrated that the DEGs were inversely correlated with TNF signaling, suggesting that the anti-tumor effects of PKU12 + siRNA treatment may be related to TNF signaling in cervical cancer. In our study, nevertheless, DEGs were inversely correlated with NF-kappa B signaling, suggesting that the anti-tumor effects of PKU12 + siRNA treatment may be mediated by suppressing NK-kappa B signaling in cervical cancer. NF-kB plays a key role in the innate and adaptive immune responses of the host. The HPV-16 E6 and E7 proteins can regulate NF-kB expression. Studies found that HPV E6 increased the expression of functional components of the NF-kappa B signaling pathways and enhanced NF-kB DNA binding activity, which was associated with an increase in pro-inflammatory cytokines (25). However, there are still controversial reports regarding the HPV16 proteins’ role in regulating NF-kappa B (26). In cancer research, ferroptosis and other regulated cell death types, including apoptosis, pyroptosis, and necroptosis, are the most relevant regulated cell death types reported in the context of cancer. Ferroptosis is characterized by iron accumulation and lipid peroxidation that ultimately induce oxidative damage (27). Wang et al. showed that persistent ferroptosis promoted cervical squamous intraepithelial lesion development and oncogenesis by regulating KRAS expression in patients with high-risk HPV infection (27). Studies also found that oleanolic acid inhibits cervical cancer HeLa cell proliferation through modulation of the ACSL4 ferroptosis signaling pathway (28). Yang et al. demonstrated that the expression profiles of ferroptosis-related genes are closely related to the tumor microenvironment and the prognostic survival of patients with cervical cancer (29). Our results also demonstrated that the DEGs were inversely correlated with ferroptosis, suggesting that the anti-tumor effects of PKU12 + siRNA treatment may be related to ferroptosis in cervical cancer. IL-17 signaling has been shown to exert an oncogenic role in cervical cancer. Bai et al. showed that IL-17 could activate JAK2/STAT3, PI3K/Akt, and the NF-kappa B signaling pathway to promote the tumorigenesis of cervical cancer (30). IL−17A could promote angiogenesis, cell proliferation, and invasion in cervical cancer (31). In addition, Feng et al. found that IL-17A promoted the migration and invasiveness of cervical cancer cells by coordinately activating MMP expression *via* the p38/NF-kappa B signaling pathway (32).

The top five upregulated and downregulated DEGs were further subjected to survival analysis. The results showed that high expression of MYH1, MYH4, FGG, and DEPP1 was associated with shorter overall survival of patients with cervical cancer; high expression of SULT1E1, RAB3C, CXCR3, and PROX2 was associated with longer overall survival of patients with cervical cancer. Among these genes, FGG is a polypeptide chain of fibrinogen. A study showed that FGG, containing many specific binding sites, could regulate cellular adhesion and invasion (33). Recently, Zhang et al. revealed that FGG promotes migration and invasion in hepatocellular carcinoma cells (34). In addition, FGG was found to be overexpressed in the tissues and blood of hepatocellular and pancreatic cancer patients (35, 36). However, the role of FGG in cervical cancer has not been studied. Our results showed that FGG was downregulated in the tumor tissues after PKU12 + siRNA treatment, and FGG may predict poor prognosis in patients with cervical cancer, suggesting that knockdown of HPV16 oncoproteins may inhibit FGG expression in cervical cancer. Sulfotransferase family 1E member 1 (SULT1E1), known as estrogen sulfotransferase, is a key enzyme that catalyzes the sulfation of estrogen and estradiol, leading to their inactivation by inhibiting their ability to bind to the estrogen receptor (37). Xu et al. showed that SULT1E1 could inhibit cell proliferation and invasion by activating PPARγ in breast cancer (37). However, the role of SULT1E1 was not examined in cervical cancer, though high SULT1E1 levels have been found in the tumor tissues of patients with breast cancer and have been associated with a poor prognosis for breast cancer in women (38, 39). Further studies may be required to elucidate the interaction between HPV16 oncoproteins and SULT1E1 in cervical cancer. CXCR3 is a chemokine receptor that is primarily expressed on CD4+ and CD8+ T cells and, to some extent, by other cells, among them epithelial cells (40). Studies demonstrated that DPP inhibition altered the CXCR3 axis and enhanced NK and CD8+ T-cell infiltration to improve anti-PD1 efficacy in murine models of pancreatic ductal adenocarcinoma (41). Koh et al. showed that high expression of CXCR3 was associated with better survival and may be a potential prognostic factor for patients with gastric cancer (42). Murakami et al. suggested that targeting CXCR3 and CXCR4 can be a promising therapy against colorectal cancer metastasis (43). In cervical cancer, Chen et al. showed that CXCL10 produced by HPV-positive cervical cancer cells stimulated exosomal PDL1 expression by fibroblasts *via* CXCR3 and JAK-STAT pathways (44). Our results showed that CXCR3 was upregulated in the tumor tissues after PKU12 + siRNA treatment, and high expression of CXCR3 may predict better a prognosis in patients with cervical cancer, suggesting that the anti-tumor effects of knockdown for HPV16 oncoproteins may be mediated *via* upregulating CXCR3 in cervical cancer.

In this study, the control group received treatment with PKU-12 + NC siRNA. PKU-12 is developed based on TD-1, and they may have a similar mechanism of action. TD-1 primarily interacts with the C-terminus of the beta-subunit of the Na(+)/K(+)-ATPase (ATP1B1). The KEGG pathways involved in ATP1B1 include “cGMP-PKG signaling pathway,” “cAMP signaling pathway,” “Cardiac muscle contraction,” “Adrenergic signaling in cardiomyocytes,” “Insulin secretion,” “Thyroid hormone synthesis,” “Thyroid hormone signaling pathway,” “Aldosterone synthesis and secretion,” “Aldosterone-regulated sodium reabsorption,” “Endocrine and other factor-regulated calcium reabsorption,” “Proximal tubule bicarbonate reclamation,” “Salivary secretion,” “Gastric acid secretion,” “Pancreatic secretion,” “Carbohydrate digestion and absorption,” “Protein digestion and absorption,” “Bile secretion,” and “Mineral absorption.” Therefore, we assume that PKU-12 may mainly affect these pathways, which do not overlap with the 20 significantly enriched pathways obtained from our sequencing results. However, this is also a limitation of our experiment, as we lack untreated tissue (blank control) to exclude these effects. Additionally, the function of PKU12 is not yet understood, but it is developed based on TD-1, which may have a similar mechanism of action, that is interacting with the Na(+)/K(+)-ATPase to create a temporary opening in the skin barrier, allowing macromolecular drugs to enter the bloodstream. Finally, we assumed protein levels of E6, E7, and L1 were reduced when treated with siRNAs, but lack of the data of Western blot. Generally speaking, the siRNA anti-sense strand binds to the target mRNA, mRNA cleavage is induced. No translation is possible. Ni et al. reported that decreased HPV 16 E6 and E7 mRNA expression was correlated with decreased translation of the gene product when using siRNA in SiHa and CaSki cells (45).

When treating HPV-derived cancer, siRNA may be a better and safer option, as HPV is the primary cause of this cancer and its oncogene is exogenous, meaning siRNA treatment would not affect host genes. For Stage 0 and Stage I cervical cancer, local treatment may be sufficient, and PKU12-based siRNA delivery may be helpful. Although still in the proof-of-concept stage, our research group is working on creating vaginal gels using this formula.

## Conclusions

In conclusion, our results showed that PKU12-based siRNA delivery significantly repressed the mRNA expression levels of HPV16 L1, E6 and E7; and attenuated tumor growth of SiHa cells in the nude mice. Further transcriptomic analysis revealed that the anti-tumor effects of PKU12-based siRNA delivery may be associated with modulating of HIF-1 signaling, TNF signaling, NF-kappa B signaling, ferroptosis, IL-17 signaling pathways.

## Data availability statement

The original contributions presented in the study are included in the article/**Supplementary Material**. Further inquiries can be directed to the corresponding author.

## Ethics statement

The animal study was reviewed and approved by Animal Experimentation Ethics Committee of Peking University and Chinese University of Hong Kong.

## Author contributions

Conceptualization, TT; methodology, YD and YSo; software, QD; validation, YD, YSo and HL; formal analysis, YSu; investigation, YZ; resources, YZ; data curation, YD; writing—original draft preparation, YD and YSo; writing—review and editing, CW; visualization, TT; supervision, CW and TT; project administration, TT; funding acquisition, TT. All authors have read and agreed to the published version of the manuscript.
