# Supplementary material for: Anti-HPV16 oncoproteins siRNA therapy for cervical cancer using a novel transdermal peptide PKU12
Source: Front Oncol. 2023 Jun 7;13:1175958. doi: 10.3389/fonc.2023.1175958 (PMC10282752; doi:10.3389/fonc.2023.1175958)
Supplement: Supplementary file 1 [file DataSheet_1.docx]

Supplementary Materials

**Anti-HPV16 oncoproteins siRNA therapy using a novel transdermal peptide PKU12**

Yan Deng ^1#^, Yi Song ^1#^, Quan Du ^2^, Chi Chiu Wang ^1,3^, Hu Li ^4,5^, Yi Sui ^6^, Yuying Zhang ^7^, Tao Tang^7,1,4^*

Table S1 Gene Ontology for the top down/up regulated transcripts

| [GO:0000146: enables microfilament motor activity](http://amigo.geneontology.org/amigo/term/GO:0000146) |
| --- |
| [GO:0000166: nucleotide binding](http://amigo.geneontology.org/amigo/term/GO:0000166) |
| [GO:0003774: cytoskeletal motor activity](http://amigo.geneontology.org/amigo/term/GO:0003774) |
| [GO:0003779: actin binding](http://amigo.geneontology.org/amigo/term/GO:0003779) |
| [GO:0005515: enables protein binding](http://amigo.geneontology.org/amigo/term/GO:0005515) |
| GO:0003725: enables double-stranded RNA binding |
| GO:0003774: cytoskeletal motor activity |
| GO:0005102: enables signaling receptor binding |
| GO:0005198: enables structural molecule activity |
| GO:0005515: enables protein binding |
| [GO:0006914: involved in autophagy](http://amigo.geneontology.org/amigo/term/GO:0006914) |
| [GO:0010506: involved in regulation of autophagy](http://amigo.geneontology.org/amigo/term/GO:0010506) |
| [GO:0000978: enables RNA polymerase II cis-regulatory region sequence-specific DNA binding](http://amigo.geneontology.org/amigo/term/GO:0000978) |
| [GO:0001222: enables transcription corepressor binding](http://amigo.geneontology.org/amigo/term/GO:0001222) |
| [GO:0003677: enables DNA binding](http://amigo.geneontology.org/amigo/term/GO:0003677) |
| [GO:0004062: enables aryl sulfotransferase activity](http://amigo.geneontology.org/amigo/term/GO:0004062) |
| [GO:0005515: enables protein binding](http://amigo.geneontology.org/amigo/term/GO:0005515) |
| [GO:0008146: enables sulfotransferase activity](http://amigo.geneontology.org/amigo/term/GO:0008146) |
| [GO:0000166: nucleotide binding](http://amigo.geneontology.org/amigo/term/GO:0000166) |
| GO:0005525: enables GTP binding |
| GO:0005515: enables protein binding |
| GO:0004950: enables chemokine receptor activity |
| GO:0005515: enables protein binding |
| GO:0000981: enables RNA polymerase II cis-regulatory region sequence-specific DNA binding |
| GO:0000978: enables DNA-binding transcription factor activity, RNA polymerase II-specific |


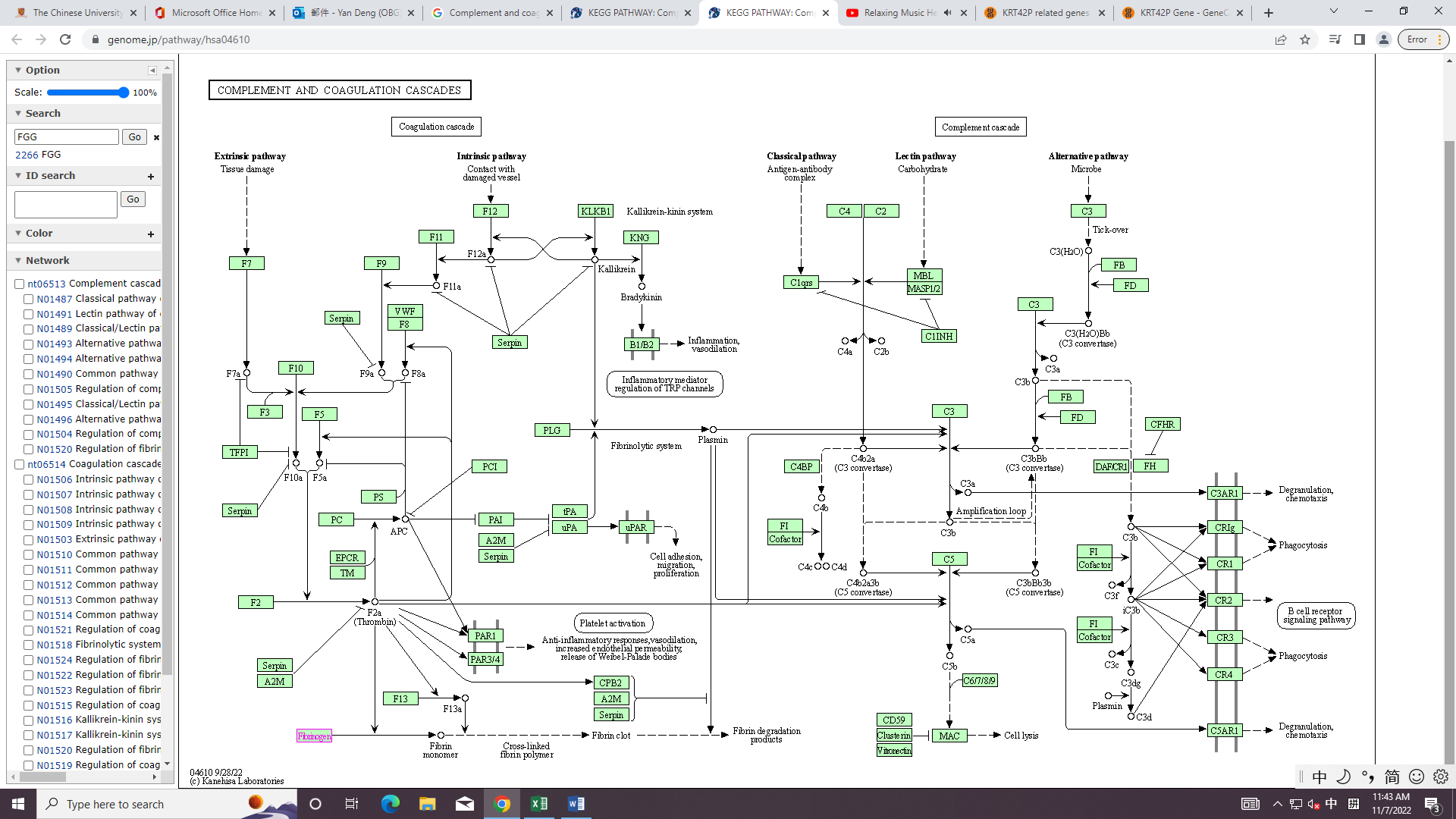


Figure S1 KEGG pathway - Complement and coagulation cascades


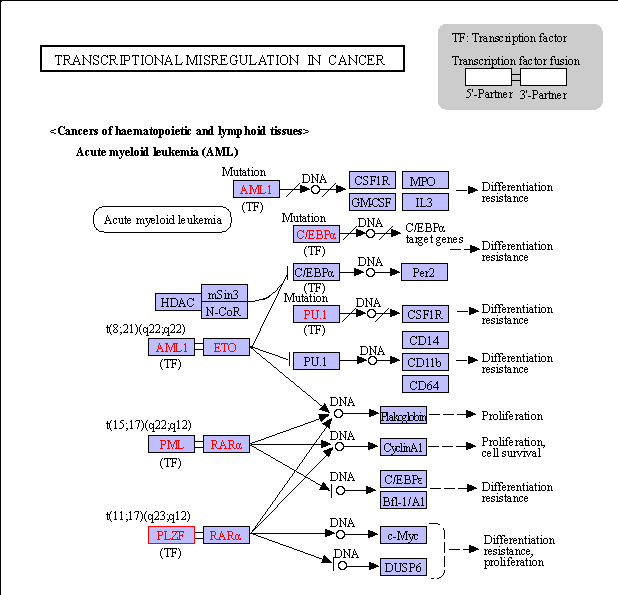


Figure S2 KEGG pathway - Transcriptional misregulation in cancer


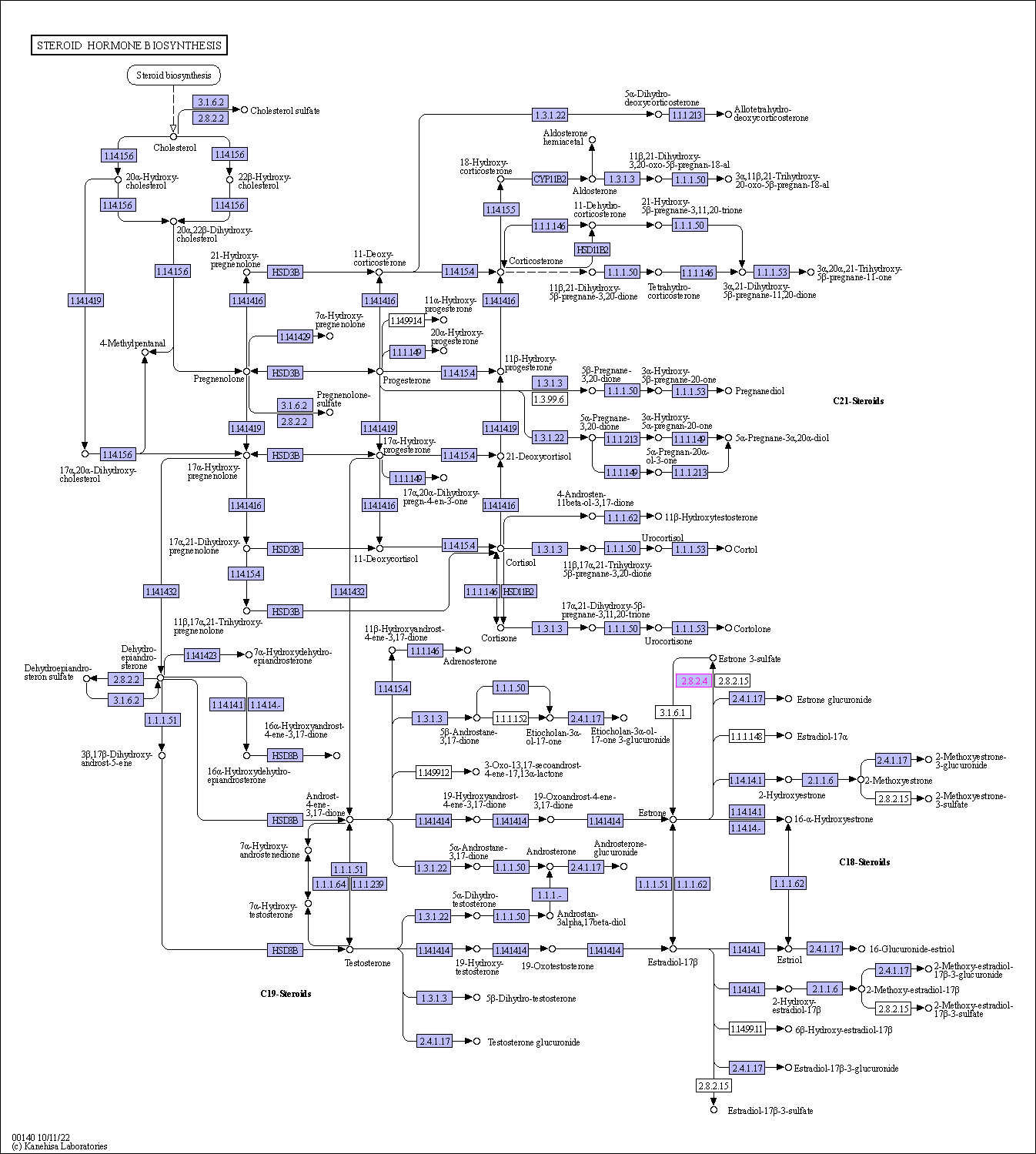


Figure S3 KEGG pathway - Steroid hormone biosynthesis


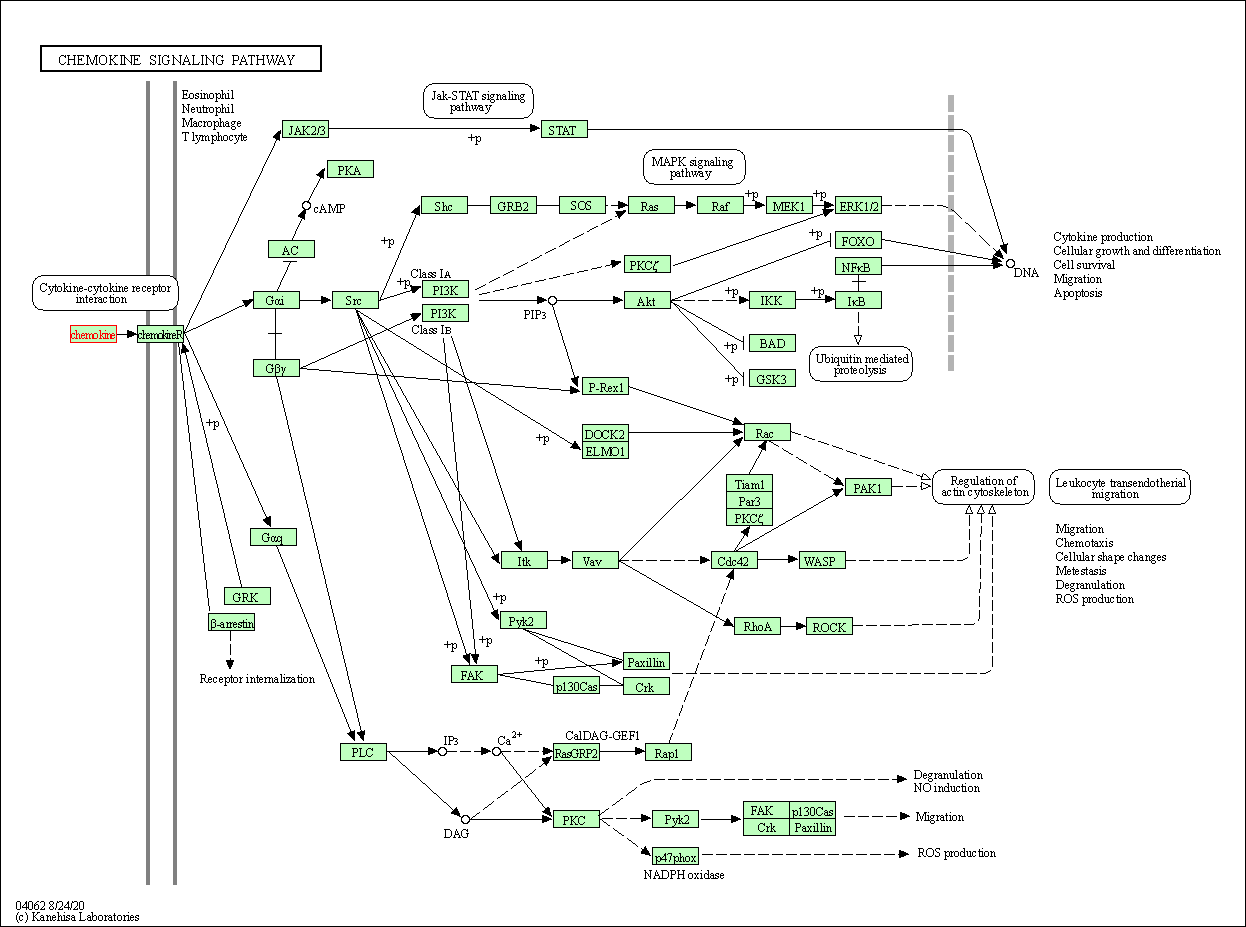


Figure S4 KEGG pathway - Chemokine signaling pathway


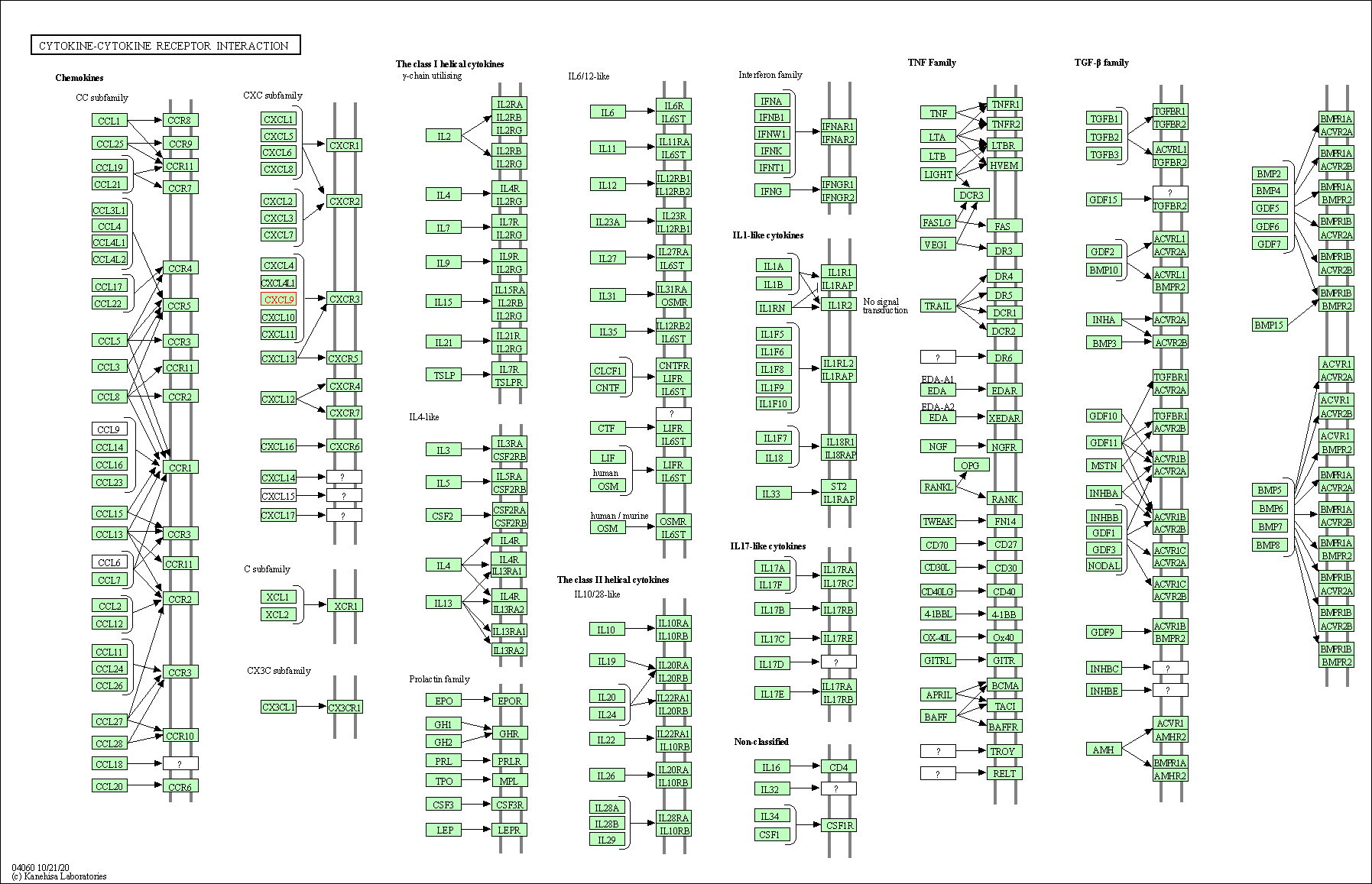


Figure S5 KEGG pathway - Cytokine-cytokine receptor interaction

Figure S6 SiRNA and PKU12 dose selection for in vivo study.

Three Balb/c athymic mice were anesthetized by Ketamine (75mg/kg) / Xylazine (10 mg/ kg) and then ten 1 cm × 1 cm area on the abdomen and the back was carefully marked. One area was used as blank, the others were applied with 1) 0.1mg PKU12+5μg GAPDH siRNA; 2) 1mg PKU12+5μg GAPDH siRNA; 3) 10mg PKU12+5μg GAPDH siRNA; 4) 0.1mg PKU12+10μg GAPDH siRNA; 5) 1mg PKU12+10μg GAPDH siRNA; 6) 10mg PKU12+10μg GAPDH siRNA; 7) 0.1mg PKU12+20μg GAPDH siRNA; 8) 1mg PKU12+20μg GAPDH siRNA; 9) 10mg PKU12+20μg GAPDH siRNA. The mice were sacrificed 24h later, the skin was cut into pieces for silencing effect measurement. As shown in the figure, 10μg siRNA/mg PKU12, 10μg siRNA/10mg PKU12, 20μg siRNA/mg PKU12 and 20μg siRNA/10mg PKU12 showed significant silence effect when compared with blank. 10μg siRNA/mg PKU12 was chosen for the further in vivo study, as its silence effect has no obvious difference comparing with the high dose groups.
